# Cell and Microvesicle Urine microRNA Deep Sequencing Profiles from Healthy Individuals: Observations with Potential Impact on Biomarker Studies

**DOI:** 10.1371/journal.pone.0147249

**Published:** 2016-01-19

**Authors:** Iddo Z. Ben-Dov, Veronica M. Whalen, Beatrice Goilav, Klaas E. A. Max, Thomas Tuschl

**Affiliations:** 1 Nephrology and Hypertension, Hadassah–Hebrew University Medical Center, Jerusalem, Israel; 2 Rockefeller University Hospital, The Rockefeller University, New York, NY, United States of America; 3 Pediatric Nephrology, Children’s Hospital at Montefiore, Albert Einstein College of Medicine, Bronx, NY, United States of America; 4 Laboratory of RNA Molecular Biology, The Rockefeller University, New York, NY, United States of America; CNRS UMR7622 & University Paris 6 Pierre-et-Marie-Curie, FRANCE

## Abstract

**Background:**

Urine is a potential source of biomarkers for diseases of the kidneys and urinary tract. RNA, including microRNA, is present in the urine enclosed in detached cells or in extracellular vesicles (EVs) or bound and protected by extracellular proteins. Detection of cell- and disease-specific microRNA in urine may aid early diagnosis of organ-specific pathology. In this study, we applied barcoded deep sequencing to profile microRNAs in urine of healthy volunteers, and characterized the effects of sex, urine fraction (cells vs. EVs) and repeated voids by the same individuals.

**Results:**

Compared to urine-cell-derived small RNA libraries, urine-EV-derived libraries were relatively enriched with miRNA, and accordingly had lesser content of other small RNA such as rRNA, tRNA and sn/snoRNA. Unsupervised clustering of specimens in relation to miRNA expression levels showed prominent bundling by specimen type (urine cells or EVs) and by sex, as well as a tendency of repeated (first and second void) samples to neighbor closely. Likewise, miRNA profile correlations between void repeats, as well as fraction counterparts (cells and EVs from the same specimen) were distinctly higher than correlations between miRNA profiles overall. Differential miRNA expression by sex was similar in cells and EVs.

**Conclusions:**

miRNA profiling of both urine EVs and sediment cells can convey biologically important differences between individuals. However, to be useful as urine biomarkers, careful consideration is needed for biofluid fractionation and sex-specific analysis, while the time of voiding appears to be less important.

## Introduction

Urine is a source of biomarkers for diseases of the kidneys and urinary tract as well as systemic disorders [1]. In addition to proteins and serum-derived metabolites, extracellular nucleic acids present in the cells and microvesicles of urine can serve biomarker purposes. RNA is present in the urine enclosed in detached cells or in extracellular vesicles (EVs) released from intact kidney and urinary tract cells. Conditions, which alter relative quantities of individual microRNAs (miRNAs) in urine, include inflammatory processes within the genitourinary tract, malignancies and infections [2].

The presence of cell-specific miRNAs in the urine may aid early diagnosis of organ-specific pathology. In this study of healthy volunteers, we examined sex differences, relationship between matched cellular and extracellular miRNA profiles and repeatability of miRNA profiles between matched consecutive urine voids.

## Materials and Methods

### Ethics statement

This study was approved by the Institutional Review Board of The Rockefeller University Hospital. Clinical data and specimens were collected after obtaining participants’ written informed consent.

### Participants and biofluid specimens

We collected urine specimens from 20 healthy volunteers, 20–30 years old [3], representative of New York City ethnic demographics. Each volunteer provided 2 urine specimens, 1–3 h apart, each at least 50 ml. Urine specimens were handled as previously described [4]. Of note, second urine voids were more dilute, as indicated by ~1.9-fold lower creatinine concentrations (p = 0.014, paired-sample *t*-test). Total RNA was extracted from 50 ml urine sediment cells and from the ensuing cell-free, ultrafiltration-retained supernatant, a ~250-fold concentrate of the cell-free urine particles and macromolecules larger than 100 kDa, thus including EV-enclosed RNA (VS2042, Vivaproducts Inc., Littleton MA) [4]. RNA was quantified using Qubit 2.0 fluorometer [5]. Small-RNA cDNA libraries were constructed with addition of external miRNA-like synthetic calibrators (2.5 attomol per ng total RNA), and sequenced, as recently described [4]. The median amounts of input RNA used for cDNA library preparation were 10.2 ng (IQR 4.2–15.1 ng) in batches of cell-derived samples and 0.49 ng (IQR 0.28–1.44 ng) in batches of EV specimens.

### Sequencing and annotation of small RNA cDNA libraries

The obtained sequence files were trimmed and split into the separate samples according to the barcode sequences. Extracted reads were assigned annotations by aligning to the genome and small-RNA databases. For miRNA annotation we used contemporary in-house definitions (see **S1 Tables**) [6].

The sequencing data discussed in this publication have been deposited in NCBI's Gene Expression Omnibus and are accessible through GEO Series accession number GSE72183 (http://www.ncbi.nlm.nih.gov/geo/query/acc.cgi?acc=GSE72183).

### Statistical analysis

Normally distributed clinical parameters were compared using unpaired or paired t-tests. Characteristics with skewed distributions were compared using Mann-Whitney, Wilcoxon signed-rank, Friedman rank sum and Kolmogorov-Smirnov non-parametric tests, as appropriate, using Bioconductor on R [7]. The 'DESeq2' package [8] was used for analysis of differential expression. Additional Bioconductor packages were used for plotting ('ggplot2' [9] and 'rggobi' [10]), PCA and correlation analysis ('stats') and unsupervised clustering and heat map generation ('pheatmap'). Classification with machine learning methods was carried out using 'MLInterfaces' [11]. Machine learning refers to a family of computational methods for analyzing multivariate datasets. Serving as a mock-up for biomarker discovery studies, we explored using such methods the possibility to classify and predict our samples based on their miRNA profiles. Attempted algorithms include neural networks, support vector machine, discrimination analyses, k-nearest neighbor classification and recursive partitioning and regression trees. Prior to statistical analysis and presentation of miRNA, except differential expression analysis, counts were transformed to log2-counts per million by the 'limma' voom function [12], following TMM normalization using 'edgeR' [13]. **S1 Text Files** include selected R scripts and related input data used in this study.

## Results and Discussion

### Total RNA yield from urine and small RNA composition analysis

To obtain miRNA profiles we processed urine cells and EVs isolated from two urine voids, taken one to three hours apart, and provided by 20 healthy young adults (**Table 1**). Women’s urine had higher total RNA content (per given volume) in both cells and EVs, compared to men (**Table 2**). Disparities in RNA content were most prominent in the first void, where inter-gender differences of 12.8-fold for cells and 4.3-fold for EVs were observed.

**Table 1:** Characteristics of study volunteers. Anthropometric and clinical characteristics of study volunteers.

	Men (n = 10)	Women (n = 10)
Age, years	27±2	26±2
Systolic BP, mmHg	115±12	106±10
Diastolic BP, mmHg	78±9	71±10
Heart rate, bpm	62±8	69±9
Height, cm	175±6	166±7
Weight, kg	77±13	64±5
Body mass index, kg/m^2^	25.1±3.6	23.3±2.4
Waist circumference, cm	86±10	75±6
Hip circumference, cm	101±10	94±6
Waist/hip ratio	0.86±0.04	0.80±0.06
Blood urea nitrogen, mg/dl	13.3±4.4	11.0±1.8
Serum creatinine, mg/dl	1.19±0.09	0.91±0.09
Serum uric acid, mg/dl	5.56±0.62	4.07±0.83
Stick urinalysis	Negative	Negative

BP, blood pressure

bpm, beats per minute.

**Table 2:** Total RNA yields in cells and extracellular vesicles. Total RNA content (ng) in study specimens by volunteer sex.

		Men		Women		
Fraction	Void	Mean	Median (IQR)	Mean	Median (IQR)	P-value^*^
Cells	1^st^	6.2	3.6 (2.1–10.7)	79.2	48.0 (19.0–120)	<0.0001
	2^nd^	11.5	6.0 (2.9–17.1)	16	12.1 (4.3–28.7)	0.579
	P-value^†^		0.241		0.074	
EVs	1^st^	0.6	0.2 (0.0–0.4)	2.6	0.9 (0.4–4.9)	0.007
	2^nd^	1.8	1.0 (0.5–2.8)	2.6	3.0 (1.0–4.2)	0.247
	P-value^†^		0.007		0.721	

EV, extracellular vesicles

IQR, 25^th^-75^th^ percentiles

*, Mann-Whitney *u*-tests comparing women to men

†, Wilcoxon signed ranks tests comparing 2^nd^ to 1^st^ void.

Barcoded small RNA cDNA libraries were prepared from 79 total RNA specimens (one volunteer provided an insufficient volume of urine for the second void EV preparation). The samples were organized in four batches, each multiplexing 19 to 20 samples (see Methods for amounts of input RNA), and sequenced on four Illumina Genome Analyzer IIx lanes. **Table A** in **S2 Tables** displays read numbers by small RNA annotation categories.

### Quality control via analysis of spike-in calibrator oligoribonucleotides

**Fig 1:**
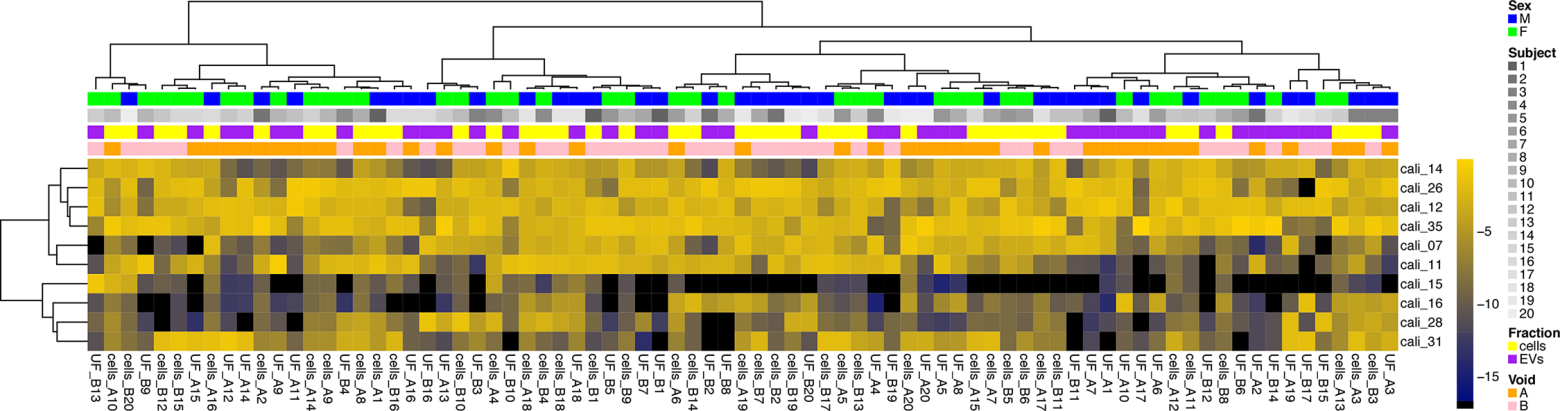
Heat map of hierarchically clustered study samples according to external calibrator content. A mixture of 10 synthetic oligoribonucleotides (‘calibrators’) was spiked into RNA samples during library preparation for sequencing. Unsupervised analysis of samples based on read counts of these calibrators shows *absence* of clustering by specimen source (cells vs. EVs), sex or void, as would be expected for an external spike-in control.

A mixture of 10 calibrator oligoribonucleotides was spiked during library preparation. Unsupervised clustering of reads corresponding to these oligonucleotides in the obtained sequence files, **Fig 1**, shows no evidence of bias related to void (first vs. second void) or sample source (cells vs. EVs). There was a trivial degree of barcode-related clustering, reflective of highly similar miRNA composition in closely related samples.

**Fig 2:**
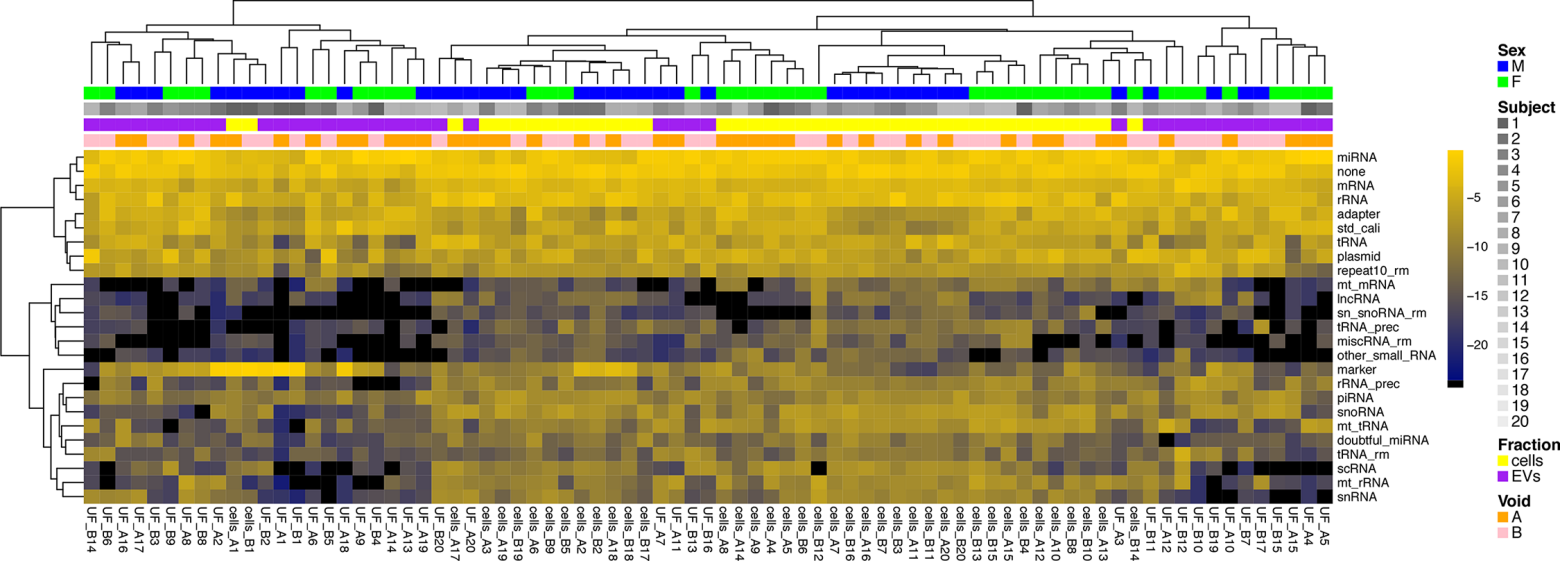
Heat map of hierarchically clustered study samples according to small RNA category profiles. Small RNA sequencing was conducted from all study samples as described in the Methods section. An automated pipeline identified and summarized the resulting sequence reads according to small RNA annotation categories. Unsupervised analysis based on read counts shows clustering by specimen source (cells vs. EVs), sex and subject.

There were no consistent sex-related or void-related differences in calibrator-normalized miRNA content. However, compared to cell-derived small RNA libraries, EV libraries were enriched with miRNA, while EV content of scRNA, snRNA, snoRNA, rRNA and rRNA precursors was lower compared to cells (Fig 2 and Table B in S2 Tables). Second and first void cells had similar small RNA composition. However, second void EVs had lower miRNA content compared to first void EVs (Table B in S2 Tables).

### Principal component analysis and unsupervised clustering

**Fig 3:**
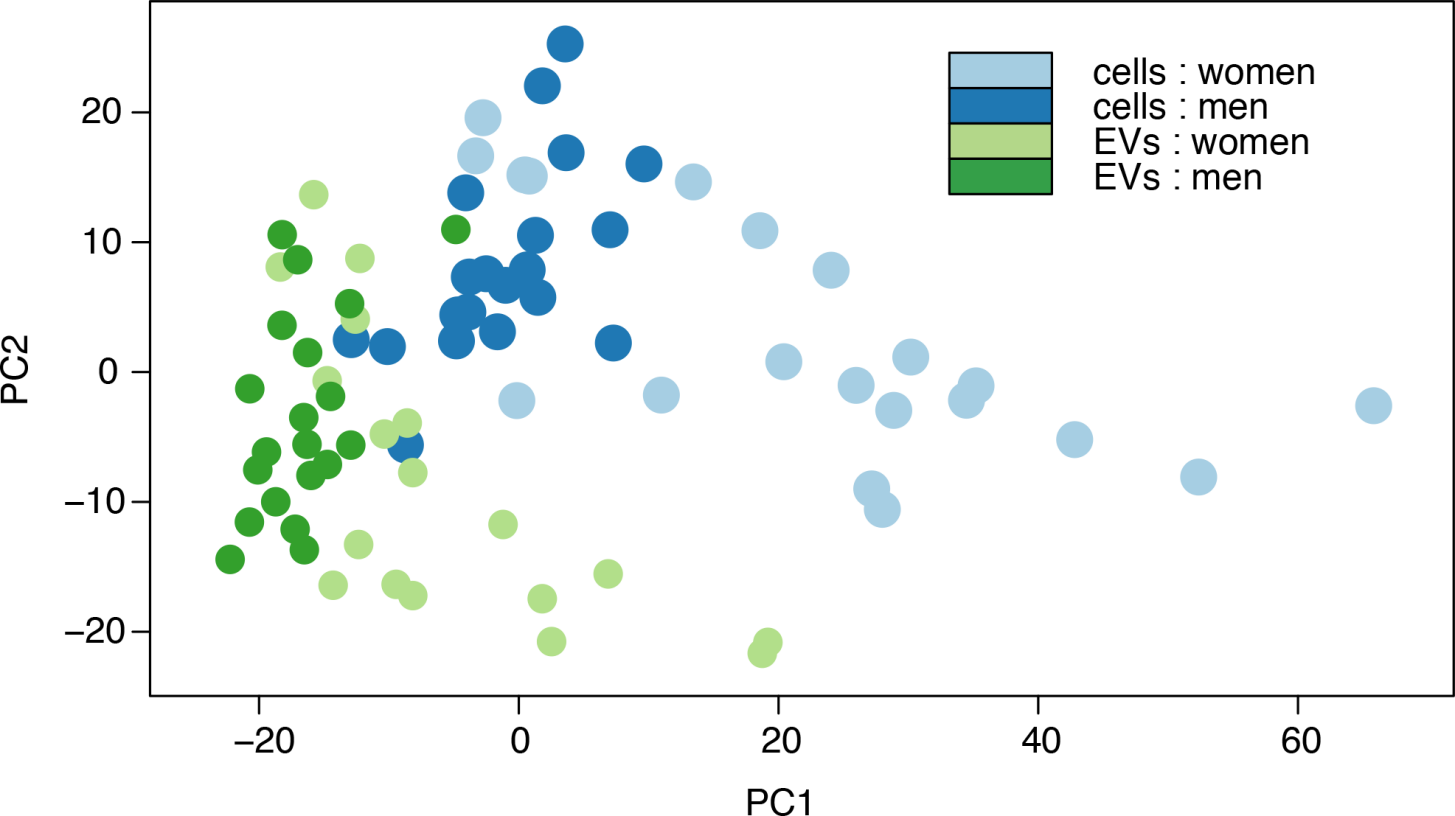
Principal component analysis plot of study sample miRNA profiles. A scatter plot depicting principle component 1 (PC1) and PC2 coordinates of study samples, color-coded according to subject sex and urine fraction. Consistent sex- and fraction-based separation of profiles is observed.

**Fig 4:**
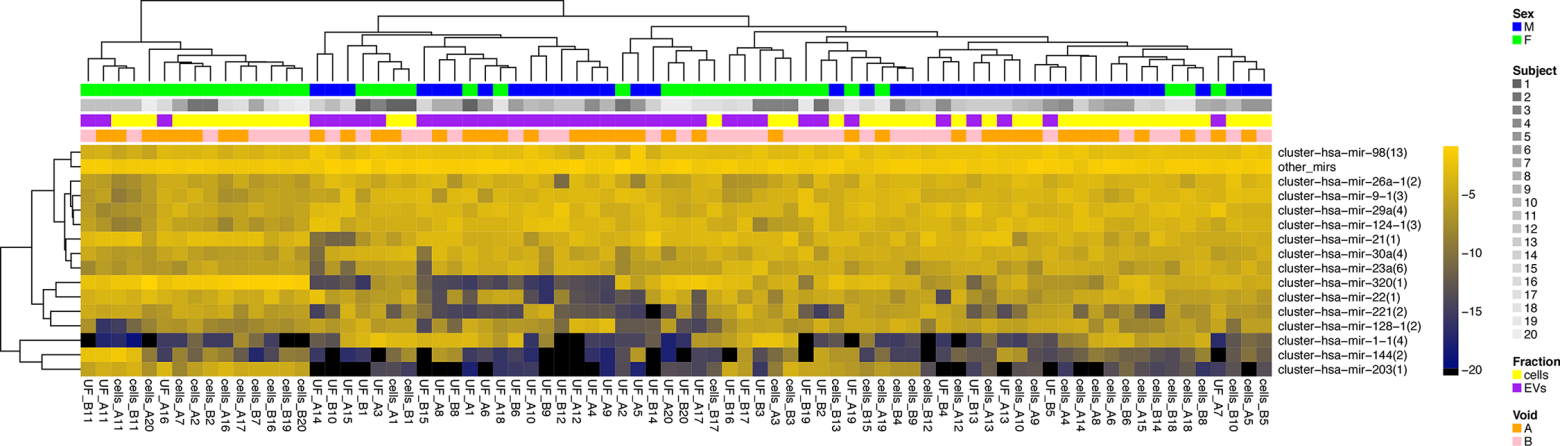
Heat map of hierarchically clustered study samples according to miRNA profiles. Small RNA sequencing was conducted from all study samples as described in the Methods section and in Figs 1 and 2. Unsupervised analysis based on miRNA precursor read counts shows prominent clustering by sex, urine fraction (cells vs. EVs) and subject.

Reads mapping to miRNA were summed, and organized according to mature miRNA (**Table C** in **S2 Tables**) and miRNA precursor clusters (**Table D** in **S2 Tables**). Principal component analysis (PCA) of mature miRNA counts, **Fig 3**, disclosed consistent sex- and fraction-based separation of profiles. Unsupervised clustering of specimens according to miRNA precursor cluster expression levels, **Fig 4**, showed prominent bundling by specimen type (cells vs. EVs) and by sex, as well as a tendency of void repeats to neighbor closely in the dendrogram. Thus, if miRNA are to be useful as urine biomarkers, careful consideration is needed for biofluid fractionation and sex-specific analysis, while the time of voiding may be less important.

### Test-retest repeatability of miRNA profiles

To examine repeatability of miRNA expression, namely the similarity of miRNA profiles in successive voids from the same individual, we examined the Spearman correlation matrix of all samples (limiting this analysis to miRNAs which were detected with ≥2 reads in ≥25% of samples– 160 mature miRNA, **Table E** in **S2 Tables**). **Table 3**shows median Spearman correlation coefficients of samples from a given batch with their *respective matched counterparts* in other batches (left panel) or with *all samples* in other batches (middle panel). miRNA profile correlations between a sample and its void (as well as fraction) counterparts are distinctly higher than correlations between miRNA profiles overall (right panel). This is another indication that intra-individual variability is considerably smaller than inter-individual differences, and that the exact timing of urine collection has minor consequences.

**Table 3:** Correlation between microRNA profiles. Median Spearman correlation coefficients between batches restricted to matched samples (left panel) or including all samples (middle panel).

	median correlation of matched samples				median correlation between all sample				KS tests-derived p-values*			
batch	EVs A	EVs B	cells A	cells B	EVs A	EVs B	cells A	cells B	EVs A	EVs B	cells A	cells B
EVs A	1	0.781	0.743	0.718	0.571	0.538	0.563	0.535	2.3E-15	3.7E-13	2.8E-11	2.6E-12
EVs B	0.778	1	0.679	0.645	0.538	0.524	0.540	0.515	3.7E-13	1.7E-14	1.3E-08	1.0E-09
cells A	0.746	0.679	1	0.733	0.563	0.540	0.581	0.554	2.8E-11	1.3E-08	2.3E-15	4.3E-13
cells B	0.722	0.645	0.733	1	0.535	0.515	0.554	0.532	2.6E-12	1.0E-09	4.3E-13	2.3E-15

EVs A, first void EVs

EVs B, second void EVs

cells A, first void sediment cells

cells B, second void sediment cells.

*, Kolmogorov-Sminrov tests comparing the lists of correlation coefficients summarized in the left panel vs. the respective lists in the middle panel.

### Analysis of differential expression

To distinguish miRNA composition in EVs vs. cells we conducted differential expression analyses using DESeq2 [8]. The expression of 129 of 431 mature miRNAs (miRNAs with at least 5 reads summed across all samples, **Table F** in **S2 Tables**) was found to differ between EVs and cells in a model including the cofactors void, sex and fraction*sex interaction. The pattern of differential expression was unbalanced in that a handful of high-abundance miRNAs were slightly enriched in EVs, while numerous low-abundance miRNA were depleted in EVs compared to cells (**Fig A** in **S1 Figs**). As an exception to this trend, the highly abundant miR-320 was less frequent in EVs. The unique biogenesis of miR-320 [14] may be linked to this relative depletion.

miRNA expression differences relating to volunteer sex (women vs. men) were evaluated in the same model. We found differential expression of 97 of 431 mature miRNA (**Table G** in **S2 Tables**). Top sex-related differentially expressed miRNAs in separate analyses on cells and EVs are shown in **Table 4**. Good agreement was found between findings in cells and EVs in respect to leading sex-related differences; Pearson correlation coefficient of log2 fold-changes among top differentially expressed miRNA in cells vs. EVs, 0.940. Thus, similar sex-related differences (and expectedly also disease-associated changes [4]) are detected in cells and EVs (see also **Fig B** in **S1 Figs**).

**Table 4:** Top differentially expressed miRNA in women compared to men. Analysis of all specimens (left), urine cells (middle) or EVs (right).

		Cells + EVs		Cells		EVs	
miRNA	CPM	log2FC	adj.pval	log2FC	adj.pval	log2FC	adj.pval
hsa-miR-320(1)	74266	3.41	5.1E-09	3.89	6.1E-06	2.93	8.1E-04
hsa-miR-21(1)	59213	0.27	5.9E-01	1.83	3.3E-03	-1.29	4.6E-02
hsa-miR-124(3)	44731	-1.33	5.9E-06	-0.63	2.4E-01	-2.03	1.0E-06
hsa-miR-29a(1)	34090	-0.92	1.1E-03	-0.14	7.9E-01	-1.71	1.1E-05
hsa-miR-9(3)	32587	-0.78	2.8E-02	-0.19	7.6E-01	-1.37	5.8E-03
hsa-miR-26a(2)	31294	-1.30	2.1E-05	-0.65	2.4E-01	-1.95	6.2E-06
hsa-miR-22(1)	30735	0.36	5.9E-01	1.82	4.2E-02	-1.10	2.7E-01
hsa-let-7f(2)	17689	-0.68	9.7E-02	-0.07	9.2E-01	-1.30	2.1E-02
hsa-miR-128(2)	14089	-1.16	2.2E-02	-0.54	5.6E-01	-1.78	1.3E-02
hsa-let-7a(3)	13876	-0.30	4.8E-01	0.46	4.9E-01	-1.06	4.4E-02
hsa-let-7c(1)	12872	-0.71	4.5E-02	-0.55	3.6E-01	-0.87	9.7E-02
hsa-miR-103(2)	10488	-0.84	8.2E-02	0.12	8.7E-01	-1.80	5.5E-03
hsa-miR-24(2)	9563	-0.88	8.0E-02	0.02	9.8E-01	-1.79	8.9E-03
hsa-miR-203(1)	8104	8.83	1.6E-32	5.80	1.3E-08	11.85	5.0E-25
hsa-miR-29b(2)	7325	-1.28	1.2E-02	-0.16	8.5E-01	-2.41	6.7E-04
hsa-miR-210(1)	6375	5.26	4.3E-14	4.57	1.0E-05	5.96	3.5E-09
hsa-miR-221(1)	6201	2.03	2.1E-03	2.64	6.5E-03	1.42	1.9E-01
hsa-let-7i(1)	5862	-0.69	2.6E-01	0.55	5.7E-01	-1.94	1.2E-02
hsa-miR-378(1)	5603	2.54	1.4E-04	2.88	3.6E-03	2.19	3.4E-02
hsa-miR-138(2)	5150	-1.33	9.8E-02	-0.38	7.8E-01	-2.29	4.2E-02
hsa-miR-205(1)	3737	9.40	1.1E-26	7.79	4.0E-12	11.01	8.3E-15

CPM, counts per million (mean normalized expression)

log2FC, log_2_ fold-change

adj.pval, adjusted p-value.

**Fig 5:**
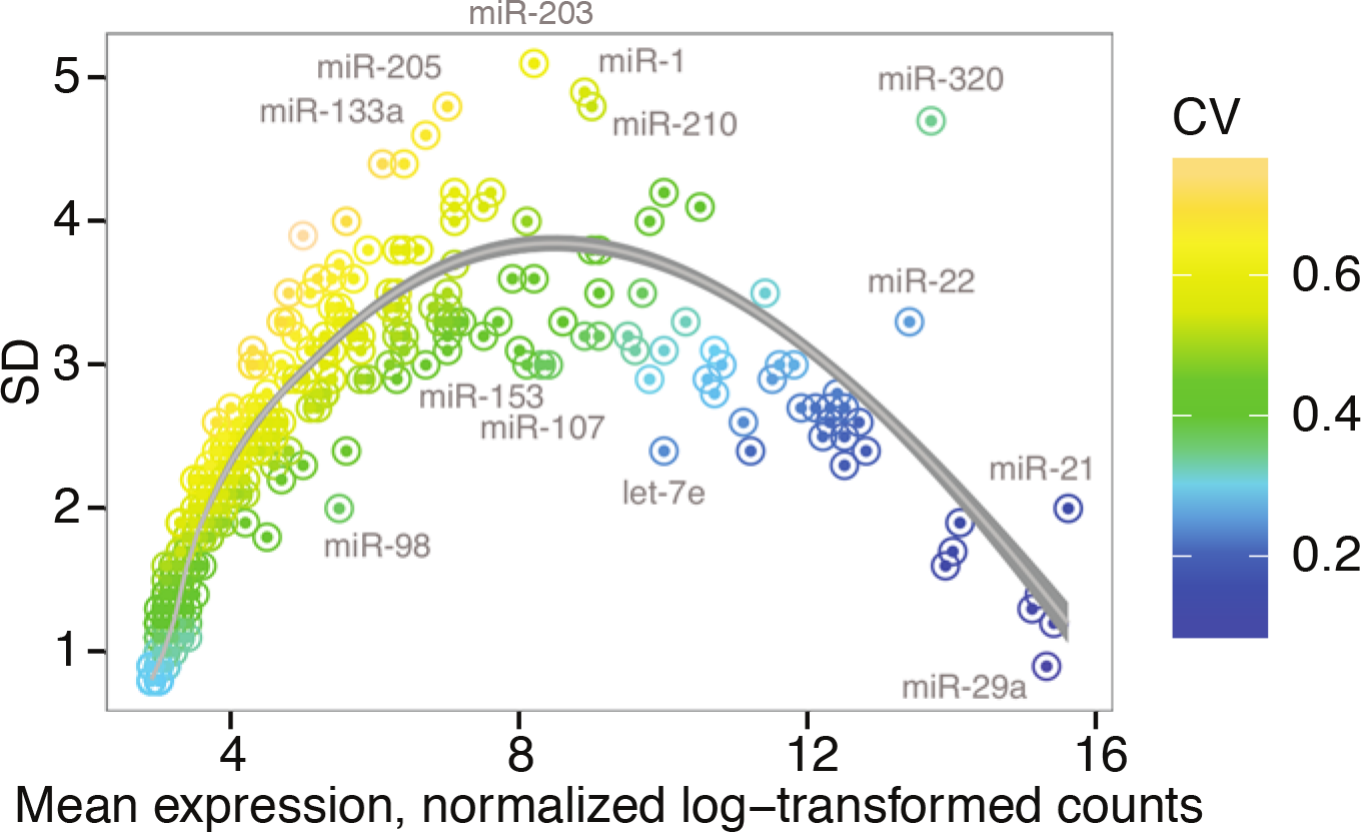
Mean-variance association of miRNA abundance. A scatter plot depicting the mean (x axis) and coefficient of variation (y axis) relationship of log-transformed normalized miRNA counts across all samples. A fit line is shown and several prominent outliers are labeled. The color scale represents the standard deviation from the mean.

**Fig 5**shows the mean-variance association of normalized and log-transformed miRNA read counts. Inverted-U-shape relationship is seen, while several outliers are denoted. This pattern would not be expected of typical tissue profiles, and is likely a result of inconsistent cell type composition, with diversity represented in the mid-abundance miRNAs. For example, miR-203 and miR-205 are expressed in keratinocytes [15], which are present in urine irregularly, predominantly in women.

### Classification

Lastly, this study involved healthy volunteers without known kidney or urinary tract disorder. However, we reasoned that in lieu of its design, this study can simulate the results of a disease-centered clinical investigation, and thus point to limitations and needed modifications to the methodology and analysis. We examined the following questions: (1) can miRNA profiles classify volunteers’ sex and specimen type, and (2) can the first void serve as a training set and guide classification (sex, specimen type) of second void specimens?

As shown above, a dendrogram (Fig 4) and a two-dimensional PCA plot (Fig 3) can partially distinguish volunteer sex and specimen fraction. Additional dimensions of data may allow better distinction. Indeed, two-dimensional projections of a multi-dimension plot are more effective at separating specimens (**Fig C** in **S1 Figs**) [16].

Various machine learning algorithms [11] performed reasonably well in classification and prediction of sample sex and fraction (**Table H** in **S2 Tables** and **Fig D** in **S1 Figs**). Using first void profiles for training, test classification of second void specimens was variably successful (**Table I** in **S2 Tables**).

## Conclusions

From this pilot study applying next generation sequencing to profile urine miRNA in healthy volunteers we conclude that miRNA profiling of both EVs and sediment cells relate to biological characteristics of interest. We found indications that intra-individual variability is considerably smaller than inter-individual differences, and that the exact timing of urine collection has minor consequences. Thus, for miRNA profiling to be useful as urine biomarkers, careful consideration is needed for biofluid fractionation and sex-specific analysis, while the time of voiding may be less important. We found similar miRNA profile differences between men and women in urine cells and extracellular vesicles, likely reflecting that extracellular vesicles are originating from the same cell types that are present in the sediment. This suggests that potential biomarkers found in urine sediment cells are likely to be present in the extracellular vesicles, and vice versa. However, in patients with kidney or urinary tract disease, as opposed to the healthy subjects who volunteered to this study, the composition of cells and extracellular vesicles in the urine may have different derivations.

## Supporting Information

S1 Figs Figs presenting supplementary data. Fig A–MA plot of miRNA expression in urine EVs compared to cells. Log fold-change (y axis) vs. average expression (x axis) in urine EVs compared to cells. Dots representing differentially expressed miRNA (adjusted p-value <0.05 according to a DESeq2 analysis) are colored red. Fig B–Sex-related fold-change values in extracellular vesicles vs. cells. Scatter plot of log_2_ fold-change values of miRNA expression in women compared to men in extracellular vesicles (EV, y axis) vs. sediment cells (x axis). Colors code the average miRNA abundance. Fig C–Multidimensional scaling of samples according to miRNA profiles. Two-dimensional projections of multidimensional scaling analysis of samples based on miRNA profiles. Principle component analysis and plotting were generated using rggobi [16]. Between 3 and 5 principal components are projected. Orange and purple, symbolize female; red and yellow, male; orange and yellow, cells; purple and red, EVs. Various projections capture clear separation based on subject sex and urine fraction. Panels b and c uncover subject 11 samples, particularly her cell specimens, as outlier. Asymptomatic bacteriuria (*E*. *coli*) is likely responsible for this aberration. Fig D–miRNA-based classification trees. Two proposed classification trees to categorize samples according to volunteer sex and specimen type (urine cells or EVs). Binary decisions are based on the specified miRNA levels (expressed as log-transformed counts per million).
(PDF)

S1 Tables Spreadsheet tables presenting miRNA annotation definition (XLSX)

S2 Tables Spreadsheet tables presenting supplementary data. (XLSX)

S1 Text Files R scripts and related input data files that can be used to reproduce the main analyses presented in this study. (ZIP)
