# Supplementary material for: Cell and Microvesicle Urine microRNA Deep Sequencing Profiles from Healthy Individuals: Observations with Potential Impact on Biomarker Studies
Source: PLoS One. 2016 Jan 19;11(1):e0147249. doi: 10.1371/journal.pone.0147249 (PMC4718679; doi:10.1371/journal.pone.0147249)
Supplement: S1 Figs — Fig A–MA plot of miRNA expression in urine EVs compared to cells. Log fold-change (y axis) vs. average expression (x axis) in urine EVs compared to cells. Dots representing differentially expressed miRNA (adjusted p-value <0.05 according to a DESeq2 analysis) are colored red. Fig B–Sex-related fold-change values in extracellular vesicles vs. cells. Scatter plot of log2 fold-change values of miRNA expression in women compared to men in extracellular vesicles (EV, y axis) vs. sediment cells (x axis). Colors code the average miRNA abundance. Fig C–Multidimensional scaling of samples according to miRNA profiles. Two-dimensional projections of multidimensional scaling analysis of samples based on miRNA profiles. Principle component analysis and plotting were generated using rggobi [16]. Between 3 and 5 principal components are projected. Orange and purple, symbolize female; red and yellow, male; orange and yellow, cells; purple and red, EVs. Various projections capture clear separation based on subject sex and urine fraction. Panels b and c uncover subject 11 samples, particularly her cell specimens, as outlier. Asymptomatic bacteriuria (E. coli) is likely responsible for this aberration. Fig D–miRNA-based classification trees. Two proposed classification trees to categorize samples according to volunteer sex and specimen type (urine cells or EVs). Binary decisions are based on the specified miRNA levels (expressed as log-transformed counts per million). (PDF) [file pone.0147249.s001.pdf]

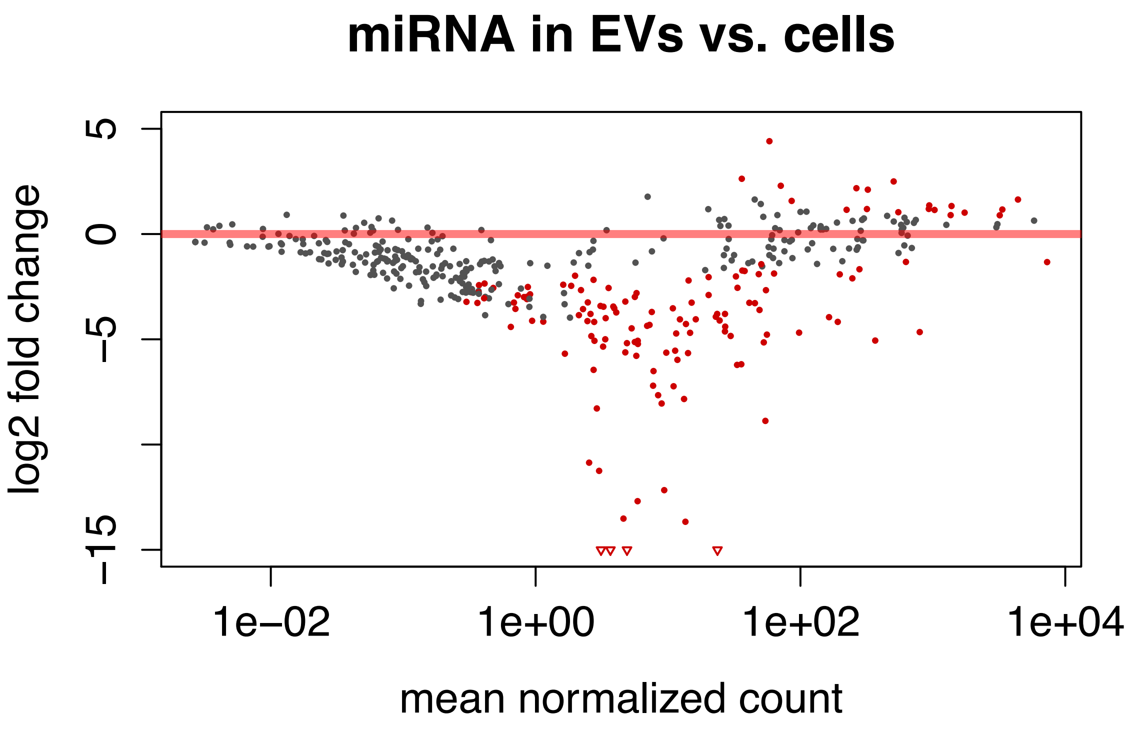

Figure A - MA plot of miRNA expression in urine EVs compared to cells

Log fold-change (y axis) vs. average expression (x axis) in urine EVs compared to cells. Dots representing differentially expressed miRNA (adjusted p-value <0.05 according to a DESeq2 analysis) are colored red.

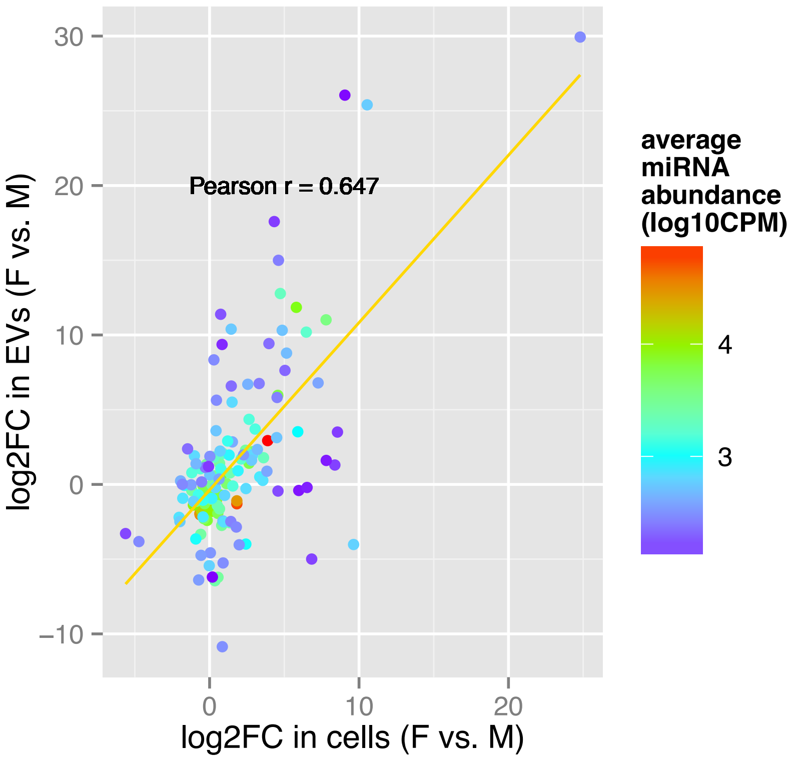

Figure B - Sex-related fold-change values in extracellular vesicles compared to cells

Scatter plot of log2 fold-change values of miRNA expression in women compared to men in extracellular vesicles (EV, y axis) vs. sediment cells (x axis). Colors code the average miRNA abundance.

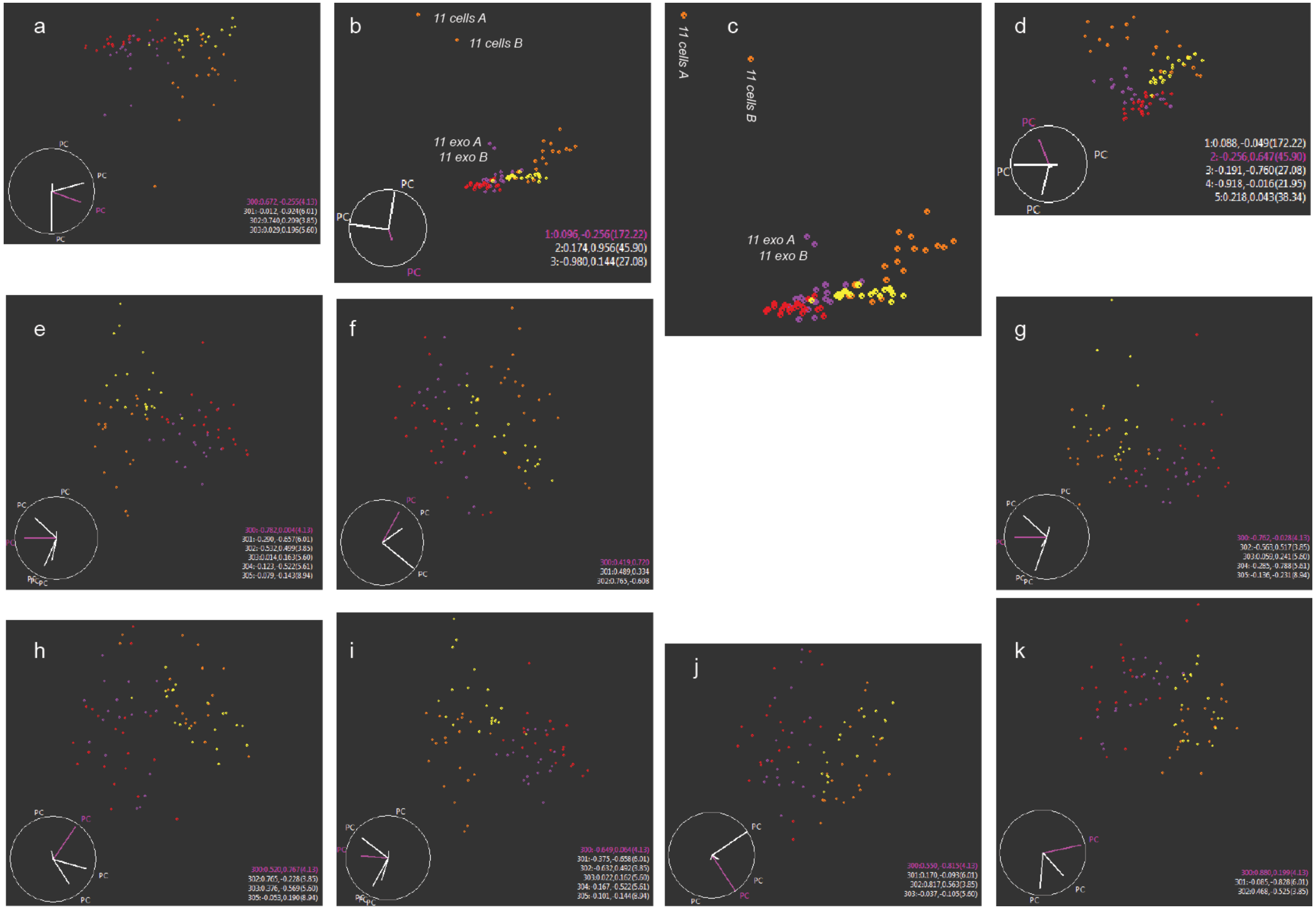

Figure C -Multidimensional scaling of samples according to miRNA profiles

Two-dimensional projections of multidimensional scaling analysis of samples based on miRNA profiles. Principle component analysis and plotting were generated using rggobi [8]. Between 3 and 5 principal components are projected. Orange and purple, symbolize female; red and yellow, male; orange and yellow, cells; purple and red, EVs. Various projections capture clear separation based on subject sex and urine fraction. Panels b and c uncover subject 11 samples, particularly her cell specimens, as outlier. Asymptomatic bacteriuria (E. coli) is likely responsible for this aberration.

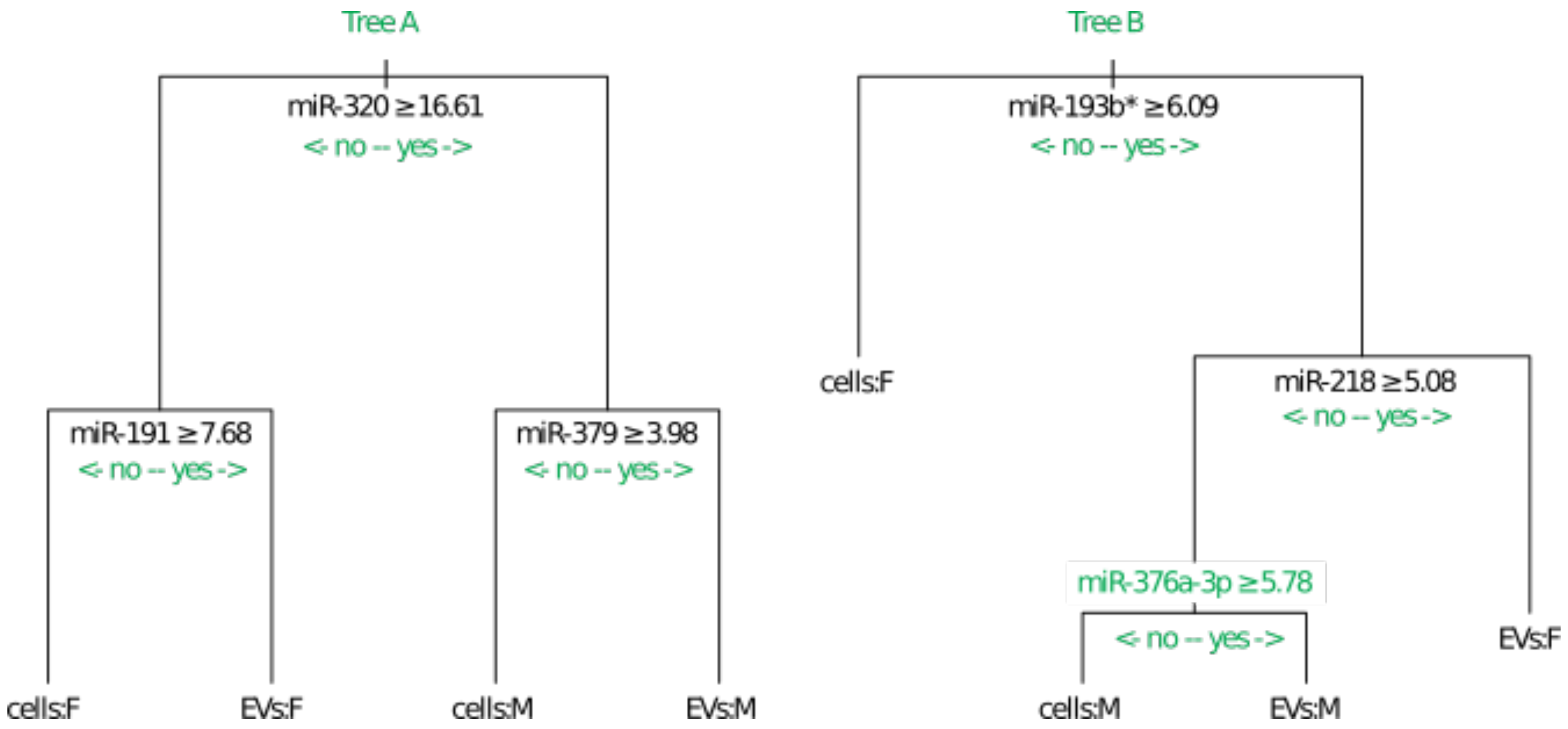

Figure D - miRNA-based classification trees

Two proposed classification trees to categorize samples according to volunteer sex and specimen type (urine cells or EVs). Binary decisions are based on the specified miRNA levels (expressed as log-transformed counts per million).
